# Correction: Impact of long-term transmural plastic stents on recurrence after endoscopic treatment of walled-off pancreatic necrosis

**DOI:** 10.1055/a-2415-7585

**Published:** 2024-09-18

**Authors:** David M de Jong, Pauline M. C. Stassen, Ivo G. Schoots, Robert C. Verdonk, Marco J. Bruno, Rogier P. Voermans, Pieter Jan F. de Jonge

**Affiliations:** 1 Gastroenterology and Hepatology, Erasmus MC University Medical Center, Rotterdam, Netherlands; 2 Radiology, Erasmus MC University Medical Center, Rotterdam, Netherlands; 3 Gastroenterology and Hepatology, St. Antonius Hospital, Nieuwegein, Netherlands; 4 Gastroenterology and Hepatology, Amsterdam University Medical Center, Amsterdam, Netherlands



In the above-mentioned article Figure 1 has been corrected. Correct is that the PFC reurrence for “Stent removal after a median of 3.4 months” is “n = 39; 25.5%”. This was corrected in the online version on September 18, 2024.

